# The planar cell polarity protein VANG-1/Vangl negatively regulates Wnt/β-catenin signaling through a Dvl dependent mechanism

**DOI:** 10.1371/journal.pgen.1007840

**Published:** 2018-12-07

**Authors:** Remco A. Mentink, Lorenzo Rella, Tomasz W. Radaszkiewicz, Tomáš Gybel, Marco C. Betist, Vitězslav Bryja, Hendrik C. Korswagen

**Affiliations:** 1 Hubrecht Institute, Royal Netherlands Academy of Arts and Sciences and University Medical Center Utrecht, Utrecht, The Netherlands; 2 Institute of Experimental Biology, Faculty of Science, Masaryk University, Brno, Czech Republic; 3 Department of Cytokinetics, Institute of Biophysics, Academy of Sciences of the Czech Republic, Brno, Czech Republic; University of California San Diego, UNITED STATES

## Abstract

Van Gogh-like (Vangl) and Prickle (Pk) are core components of the non-canonical Wnt planar cell polarity pathway that controls epithelial polarity and cell migration. Studies in vertebrate model systems have suggested that Vangl and Pk may also inhibit signaling through the canonical Wnt/β-catenin pathway, but the functional significance of this potential cross-talk is unclear. In the nematode *C*. *elegans*, the Q neuroblasts and their descendants migrate in opposite directions along the anteroposterior body axis. The direction of these migrations is specified by Wnt signaling, with activation of canonical Wnt signaling driving posterior migration, and non-canonical Wnt signaling anterior migration. Here, we show that the Vangl ortholog VANG-1 influences the Wnt signaling response of the Q neuroblasts by negatively regulating canonical Wnt signaling. This inhibitory activity depends on a carboxy-terminal PDZ binding motif in VANG-1 and the Dishevelled ortholog MIG-5, but is independent of the Pk ortholog PRKL-1. Moreover, using Vangl1 and Vangl2 double mutant cells, we show that a similar mechanism acts in mammalian cells. We conclude that cross-talk between VANG-1/Vangl and the canonical Wnt pathway is an evolutionarily conserved mechanism that ensures robust specification of Wnt signaling responses.

## Author summary

Wnt proteins are signaling molecules with a wide range of functions in embryonic development and the maintenance of adult tissues. Wnt proteins can trigger several different signaling pathways that are grouped in β-catenin dependent (canonical) and independent (non-canonical) signaling mechanisms. Here, we have investigated cross-talk between these different Wnt signaling pathways. We show that VANG-1/Vangl, a component of the non-canonical planar cell polarity pathway, negatively regulates canonical Wnt signaling. We propose that this cross-talk mechanism ensures that Wnt stimulated cells always activate the proper downstream signaling response.

## Introduction

Wnt proteins are members of an evolutionarily conserved family of secreted signaling molecules that play a central role in development, adult tissue homeostasis and disease [1, 2]. Wnt proteins can trigger a variety of responses in target cells–including cell fate specification, cell polarization and migration–that are mediated through distinct canonical and non-canonical Wnt signal transduction pathways [3].

The pathway that has been studied in most detail is the canonical Wnt pathway, which controls target gene expression through the effector protein β-catenin [1, 3]. In the absence of Wnt signaling, β-catenin is targeted for proteasomal degradation by a destruction complex that consists of the scaffold protein Axin, the tumor suppressor gene product APC and the protein kinases CK1 and GSK3β. Binding of Wnt to the receptors Frizzled and LRP6 leads to inhibition of β-catenin degradation through a mechanism that involves the cytoplasmic protein Dishevelled (Dvl) and recruitment of Axin to LRP6 at the plasma membrane. This enables the stabilized β-catenin to translocate to the nucleus, where it interacts with TCF/Lef1 transcription factors to co-activate target gene transcription.

Non-canonical Wnt signaling occurs independently of β-catenin and comprises several different pathways that directly influence cytoskeletal dynamics to control cell polarity or migration [3]. One of these non-canonical Wnt pathways is known as the planar cell polarity (PCP) pathway, which is required in *Drosophila* and vertebrates for the correct polarization of epithelial cells along the plane of the epithelial tissue [4]. The core components of this pathway are the trans-membrane proteins Frizzled and Van Gogh-like (Vangl) and the cytoplasmic proteins Dvl and Prickle (Pk), which drive planar polarization by asymmetrically localizing to the proximal (Vangl and Pk) and distal side (Frizzled and Dvl) of epithelial cells. How this asymmetric localization of PCP pathway components is established and which role Wnt ligands play in this process is still poorly understood.

In addition to planar cell polarity, Vangl and Pk also have other functions. Both are required for cell migration during vertebrate development. Examples are the convergence and extension cell movements during gastrulation in zebrafish and *Xenopus* [5–7], cell movements during neurulation and neural tube formation [8, 9] and the migration of motoneurons during brain development [5, 10]. Furthermore, several lines of evidence suggest that there may also be an antagonistic relationship between Vangl and Pk and the canonical Wnt/β-catenin pathway. Overexpression of Vangl2 or Pk has been shown to reduce canonical Wnt signaling activity in reporter gene assays [11, 12]. In vivo experiments in zebrafish have demonstrated that a decrease in Vangl2 activity enhances the dorsalizing effect of Wnt8a [13] and induces a low frequency of embryos with a duplicated body axis [14], both hallmarks of overactive canonical Wnt signaling. Finally, Vangl2 has been implicated in the Wnt5a dependent inhibition of canonical Wnt signaling during mouse limb development [15].

To further study the potential cross-talk between Vangl and Pk and the canonical Wnt pathway, we turned to the nematode *C*. *elegans*, which offers a more simplified Wnt signaling system with only 5 Wnt ligands, 4 Frizzled receptors and single orthologs of Vangl and Pk that control the migration and polarity of defined cells and axons [16–22]. Among the cells that respond to Wnt ligands are the two Q neuroblasts and their descendants [18, 23]. QL and QR are born at similar positions on the left and right lateral side of the animal, but migrate in opposite directions: QL and its descendants migrate posteriorly, while QR and its descendants migrate towards the anterior [24]. The difference in migration direction is specified by distinct responses of QL and QR to the Wnt ligand EGL-20 [25]. EGL-20 activates a canonical Wnt/β-catenin pathway in QL that leads to the expression of the homeotic gene *mab-5* [26–29], which in turn directs the migrating QL descendants towards the posterior [30, 31]. QR also responds to EGL-20/Wnt, but activates a non-canonical Wnt signaling mechanism that induces migration of the QR descendants towards the anterior [20, 32]. Importantly, both QL and QR can activate canonical Wnt signaling, but a difference in response threshold ensures that under normal conditions only QL activates *mab-5* expression [25]. When EGL-20/Wnt is overexpressed or when negative regulators such as the Axin ortholog *pry-1* are mutated, both QL and QR activate canonical Wnt signaling and *mab-5*/Hox expression, and as a consequence, both the QL and QR descendants migrate towards the posterior. Conversely, in mutants that disrupt canonical Wnt signaling, *mab-5* fails to be expressed in QL and the QL descendants localize to similar anterior positions as the QR descendants. The final position of the Q neuroblast descendants therefore provides a sensitive assay to study the interplay between canonical and non-canonical Wnt signaling mechanisms.

We have previously shown that the *C*. *elegans* orthologs of Vangl and Pk, VANG-1 and PRKL-1, are part of a non-canonical Wnt signaling pathway that controls the short-range anteroposterior and dorsoventral migration of the final QR descendants QR.paa and QR.pap [32]. Here, we show that in addition to this direct role in cell migration, VANG-1 also functions as a negative regulator of the canonical Wnt pathway dependent expression of *mab-5*/Hox in the Q neuroblast lineage. We demonstrate that VANG-1 acts independently of PRKL-1, through a mechanism that requires the carboxy-terminal PDZ binding domain that binds the Dvl ortholog MIG-5, and extend these findings to mammalian Vangl and Dvl using Vangl1 and Vangl2 double knock out cells. Our results provide further evidence for an evolutionarily conserved function of Vangl as a negative regulator of canonical Wnt signaling, and have important implications for the potential role of Vangl as a tumour suppressor gene.

## Results

### Simultaneous loss of *vang-1* and *sfrp-1* leads to ectopic activation of canonical Wnt/β-catenin signaling and *mab-5* expression in the QR lineage

**Fig 1:**
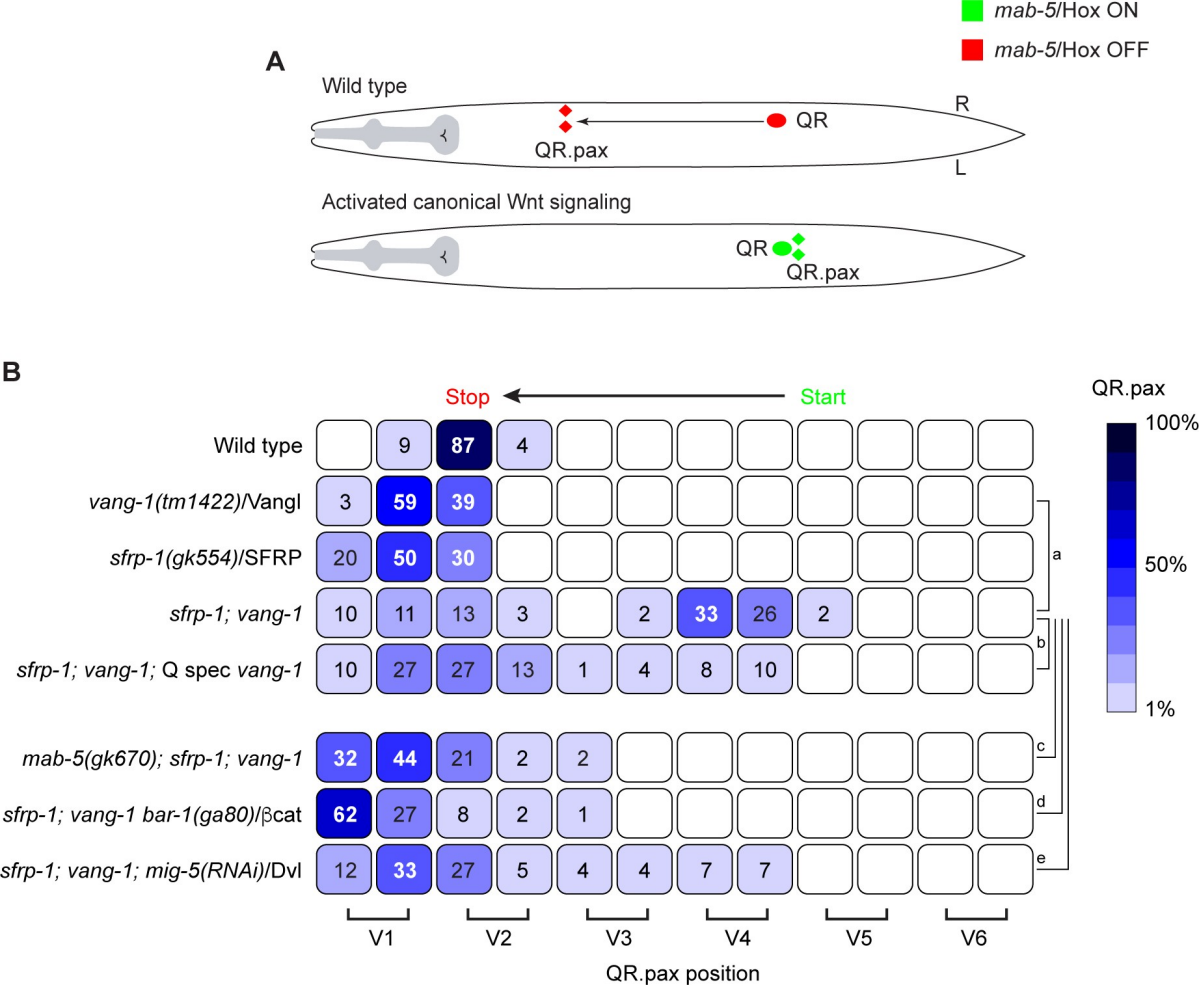
Loss of *vang-1*/Vangl and *sfrp-1*/SFRP leads to ectopic activation of canonical Wnt/β-catenin signaling in the QR descendants. **(A)** Schematic representation of QR descendant migration. In wild type animals, *mab-5*/Hox is not expressed in QR (red) and the QR descendants migrate anteriorly. A gain of canonical Wnt signaling leads to expression of *mab-5* (green) and posterior localization of the QR descendants **(B)** Final positions of the QR.pax with respect to the seam cells V1.a to V6.p (lower brackets indicate Vn.a (left) and Vn.p (right) daughters of Vn cells). Values listed are the cumulative percentiles of the total number of cells scored in 3 independent experiments, n>30 for each experiment. The *egl-17* promoter was used to specifically express wild type *vang-1* in the Q lineage (Q spec *vang-1*). A color (blue) coded heat map represents the range of percentile values. Statistical significance was calculated using a Student’s t-test (a, p<0.01; b, c, d, p<0.001; e, p <0.0001). For details on statistics see materials and methods.

A first indication of a role for *vang-1* in canonical Wnt signaling came from our analysis of double mutants between *vang-1* and *sfrp-1* (Fig 1A and 1B). *sfrp-1* encodes a secreted Frizzled-related protein that functions as a negative regulator of Wnt signaling by binding and sequestering Wnt ligands [33]. Loss of *sfrp-1* results in a global increase in Wnt signaling, but is insufficient to trigger canonical Wnt signaling and *mab-5*/Hox expression in QR. However, when we combined *sfrp-1* with a mutation in *vang-1*, over 60% of the double mutants showed posterior localization of the final QR descendants QR.paa and QR.pap (abbreviated as QR.pax). Examination of *mab-5*; *sfrp-1*; *vang-1* triple mutants revealed that in the absence of *mab-5*/Hox the QR descendants no longer localized in the posterior, indicating that *vang-1* and *sfrp-1* act genetically upstream of *mab-5*.

**Fig 2:**
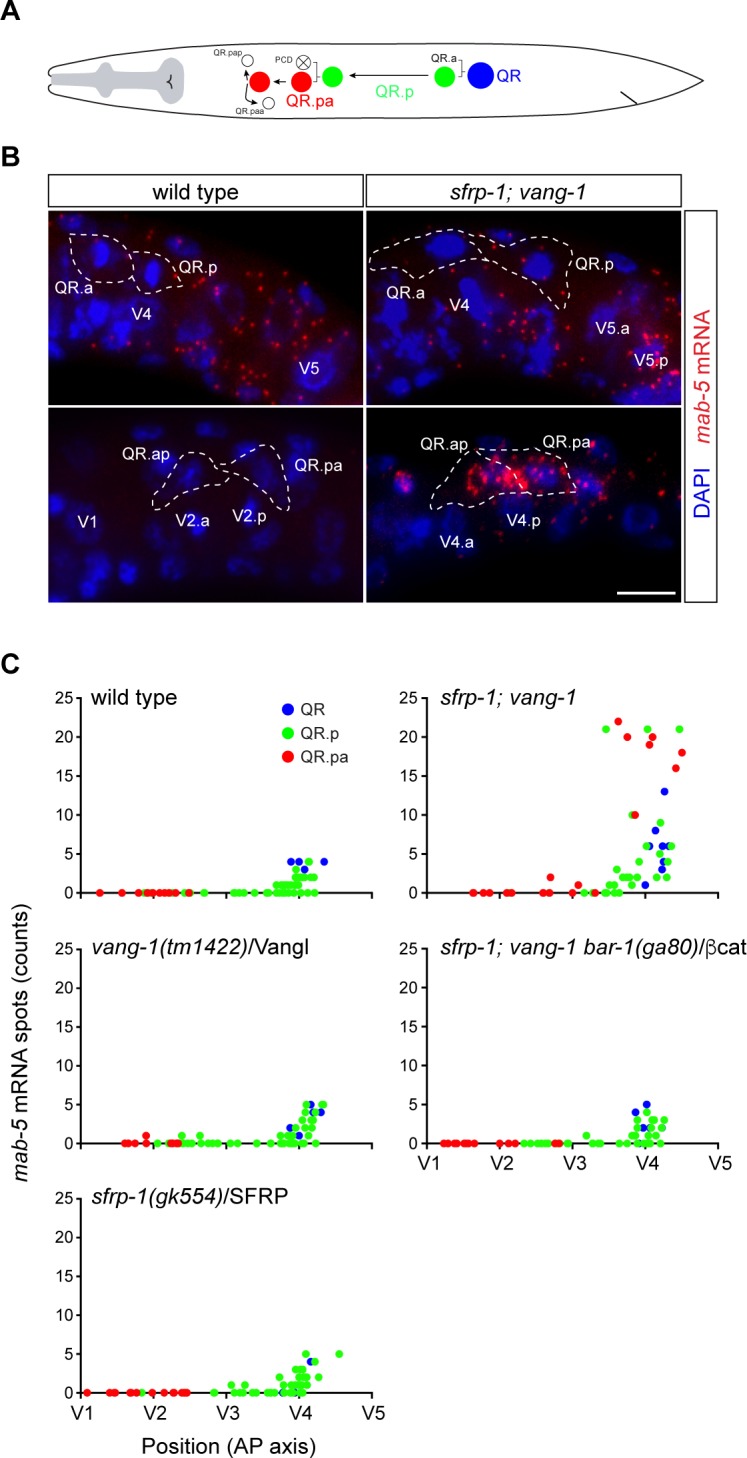
*mab-5*/Hox is ectopically expressed upon simultaneous loss of *vang-1*/Vangl and *sfrp-1*/SFRP. **(A)** Schematic overview of the anterior migration of the QR neuroblast and its descendants QR.p and QR.pa. The migration of QR.a and its descendants is not shown. Apoptotic cells are represented as white cells with a cross. Expression of *mab-5* mRNA was quantified in QR (blue), QR.p (green) and QR.pa (red). Colors correspond to those used in the graphs depicting *mab-5* transcription dynamics in panel C. **(B)** Single molecule mRNA FISH (smFISH) of *mab-5* mRNA (red) in wild type and *sfrp-1; vang-1* double mutants. A high number of *mab-5* mRNA spots can be observed in the QR.pa and QR.ap cells of *sfrp-1; vang-1* mutant animals. Q neuroblasts were identified by GFP labeling using the *heIs63* transgene. Nuclei are visualized with DAPI staining (blue). Scale bar is 5 μm. **(C)** Transcription dynamics of *mab-5* in wild type and mutant animals as quantified in QR (blue), QR.p (green) and QR.pa (red) neuroblasts, n>60 for all genotypes, representing >2 independent experiments. The number of mRNA spots per cell is plotted against the position of the cell with respect to the seam cells V1 to V5.

To investigate whether *mab-5*/Hox is ectopically expressed in *sfrp-1*; *vang-1* double mutants, we quantitatively measured *mab-5* expression in the QR lineage using a single molecule mRNA fluorescent in situ hybridization (smFISH) approach [29, 34, 35]. In wild type animals and *vang-1* or *sfrp-1* single mutants, less than five *mab-5* transcripts were detected in QR (Fig 2A and 2C). This number decreased in QR.p and no *mab-5* transcripts could be detected in QR.pa (Fig 2B and 2C). In *sfrp-1*; *vang-1* double mutants, on the other hand, there was a clear increase in *mab-5* transcript number in the QR lineage, with some animals showing over 20 *mab-5* transcripts in QR.p or QR.pa. Consistent with the role of *mab-5* in promoting posterior localization of the Q neuroblast descendants [31], high *mab-5* expression was only observed in QR.p or QR.pa cells positioned in the posterior (Fig 2C). Indeed, time-lapse live cell imaging showed that posteriorly localized QR.p cells are at this position because they fail to migrate anteriorly (S1 and S2 Movies).

In the QL lineage, the expression of *mab-5*/Hox is regulated by canonical Wnt signaling [26, 28–30]. To test whether the ectopic expression of *mab-5* in the QR lineage of *sfrp-1*; *vang-1* double mutants is also dependent on canonical Wnt signaling, we examined QR.pax position in triple mutants of *sfrp-1*, *vang-1* and the canonical β-catenin gene *bar-1*. As shown in Fig 1B, the QR.pax localized anteriorly in *sfrp-1*; *vang-1*; *bar-1* mutants. A similar result was obtained when we knocked down the Dvl ortholog *mig-5* (Fig 1B). Furthermore, there was no increase in *mab-5* expression in the *sfrp-1*; *vang-1 bar-1* triple mutant (Fig 2C). Indeed, the overall expression dynamics of *mab-5* in the QR lineage was comparable to the *sfrp-1* and *vang-1* single mutants. We conclude that the combined loss of *sfrp-1* and *vang-1* leads to activation of canonical Wnt signaling and *mab-5*/Hox expression in the QR lineage.

### *vang-1* functions cell autonomously in the Q neuroblast lineage

We have previously shown that *vang-1* is expressed in QR.p, QR.pa and the final QR descendants QR.pap and QR.paa [32]. Here, we have extended this analysis and show that *vang-1* transcripts can be detected in both the QL and QR neuroblasts at the onset of larval development (S1 Fig). During the initial polarization and migration of the Q neuroblasts that precedes their response to Wnt ligands [35, 36], there is a clear increase in *vang-1* expression in both neuroblasts to an average of 4 ± 1.5 transcripts. After division, there is a significant decrease in *vang-1* expression in QL.p (1.5 ± 1 transcripts) and QR.p (1.5 ± 1 transcripts), but this expression remains relatively constant during QR.p and QR.pa migration [32]. To investigate whether *vang-1* is required within the Q neuroblast lineage, we expressed wild type *vang-1* in *sfrp-1*; *vang-1* double mutants using the Q neuroblast specific *egl-17* promoter (Q spec *vang-1*) [37]. We found that the percentage of animals with posteriorly displaced QR.pax cells was significantly lower as in the non-transgenic siblings (Fig 1B). We conclude that *vang-1* acts cell autonomously in the Q neuroblast lineage to inhibit canonical Wnt signaling.

### *vang-1* mutants display a lower threshold for EGL-20/Wnt induced canonical Wnt/β-catenin pathway activation

**Fig 3:**
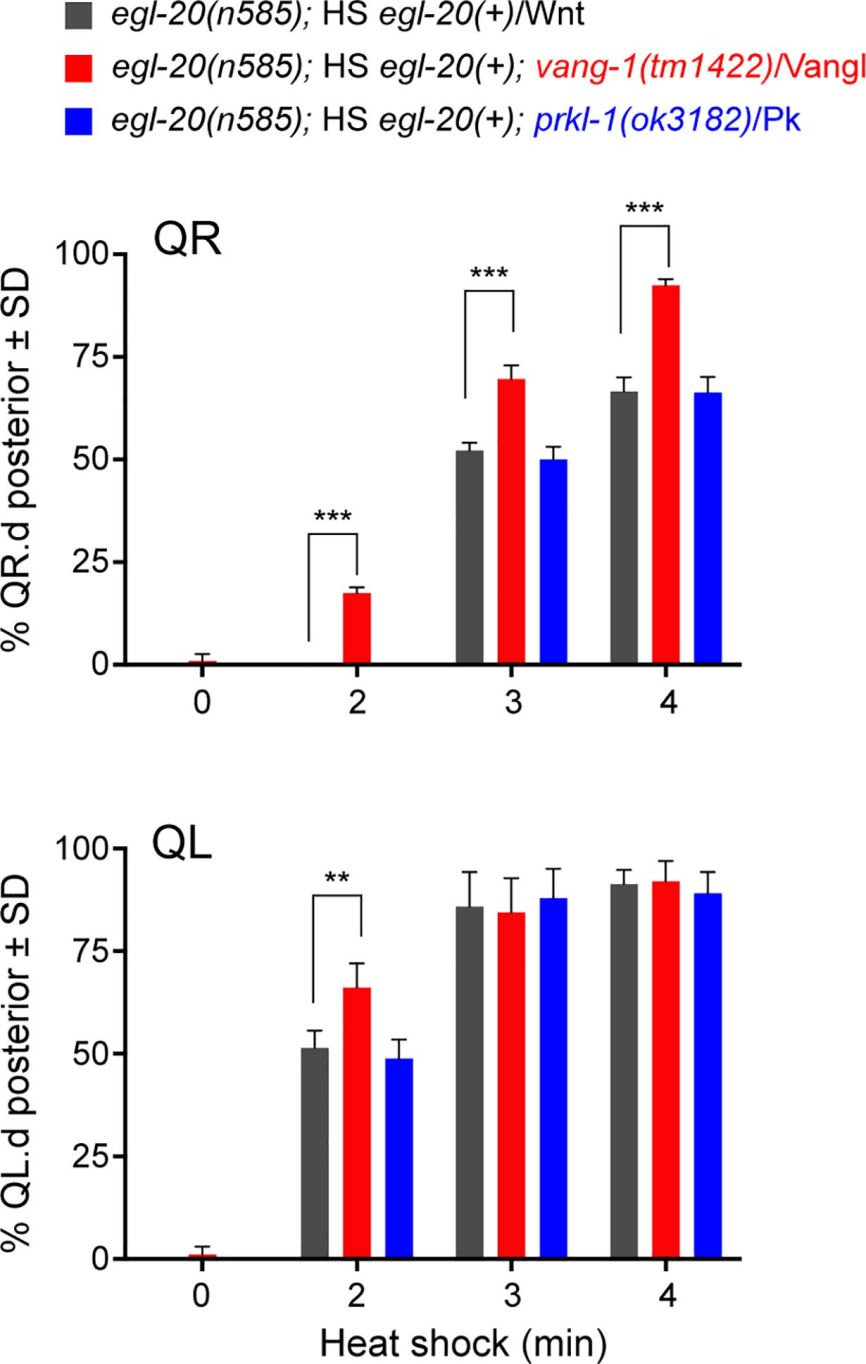
Loss of *vang-1*/Vangl lowers the threshold for EGL-20/Wnt dependent activation of canonical Wnt/β-catenin signaling in the QL and QR neuroblast lineages. Activation of canonical Wnt signaling in *egl-20* (grey), *egl-20; vang-1* (red) and *egl-20 prkl-1* (blue) mutants ectopically expressing *egl-20* using a heat shock promoter. The percentage of QR.pax cells (top graph) and QL.pax cells (lower graph) localizing in the posterior is plotted against the duration of heat shock. Quantification of posteriorly localized Q.pax cells is indicated as mean ± SD and represents the results of experiments performed in triplicate. Statistics were calculated using a Student’s t-test (** p<0.01, *** p<0.001).

Even though canonical Wnt signaling and *mab-5*/Hox expression is normally not induced in QR, the QR neuroblast can activate the pathway when the Wnt ligand EGL-20 is overexpressed [25]. As loss of *sfrp-1* has been shown to increase EGL-20/Wnt signaling activity [33], we hypothesized that loss of *vang-1* may activate *mab-5* expression in the *sfrp-1* mutant background by lowering the threshold for EGL-20/Wnt induced canonical Wnt pathway activation. To test this model, we inducibly expressed *egl-20*/Wnt using a heat shock promoter in an *egl-20* mutant background and compared the response of wild type and *vang-1* mutant animals to increasing doses of EGL-20/Wnt (Fig 3A). As expected, there was a clear correlation between the duration of heat shock and the percentage of animals with posteriorly displaced QR.pax. Consistent with our hypothesis, we found that at each of these conditions, the percentage of posteriorly displaced QR.pax was significantly higher in the *vang-1* mutant background (p<0.0001, Student’s t-test). We conclude that loss of *vang-1* lowers the threshold of QR for EGL-20/Wnt induced canonical Wnt pathway activation.

### *vang-1* negatively regulates canonical Wnt/β-catenin signaling in the QL lineage

**Fig 4:**
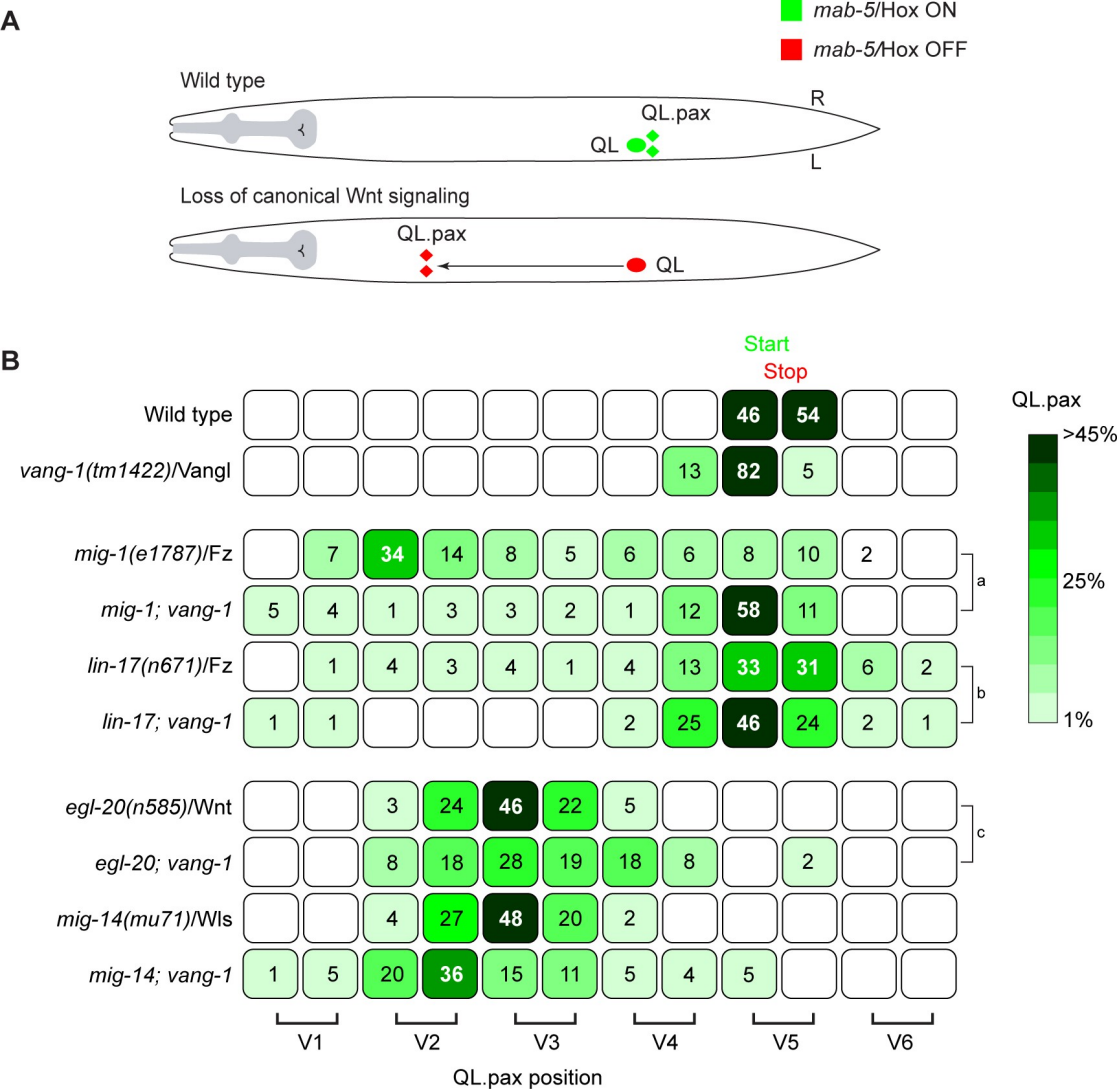
Loss of *vang-1*/Vangl restores posterior localization of the QL descendants in *mig-1* and *lin-17* Frizzled mutants. **(A)** Schematic representation of QL descendant migration. In wild type animals, *mab-5*/Hox is expressed in QL (green) and the QL descendants localize in the posterior. When canonical Wnt signaling is abrogated, *mab-5* is not expressed (red) and the QL descendants migrate anteriorly. **(B)** Final positions of the QL.pax with respect to the seam cells V1.a to V6.p (lower brackets indicate Vn.a (left) and Vn.p (right) daughters of Vn cells). Values listed are the cumulative percentiles of the total number of cells scored in at least 3 independent experiments, n>30 for each experiment. Statistical significance was calculated using a Student’s t-test (a, b, c, p<0.05).

To investigate whether *vang-1* also influences canonical Wnt signaling in the QL lineage, we asked whether loss of *vang-1* influences QL descendant migration in mutants in which canonical Wnt signaling is partially disrupted. The Frizzled receptors *mig-1* and *lin-17* are part of interlocked positive and negative feedback loops that ensure robust activation of *mab-5*/Hox expression in QL [29]. In *mig-1*/Fz mutants, *mab-5* expression is strongly reduced, and as a consequence, the QL descendants frequently migrate anteriorly (Fig 4A and 4B) [30]. A similar, but more subtle phenotype is observed in *lin-17*/Fz mutants. When we combined the *mig-1*/Fz mutant with *vang-1*, there was significant rescue of posterior QL.pax localization, a trend that was also observed in the *lin-17*; *vang-1* double mutant. The restoration of posterior QL.pax positioning suggests that loss of *vang-1* compensates for reduced Wnt/β-catenin signaling activity and is consistent with a general negative regulatory role of *vang-1* in the Q neuroblast lineage. This conclusion is further supported by our finding that in *vang-1* mutants, QL showed an enhanced canonical Wnt signaling response to ectopically expressed EGL-20/Wnt (Fig 3B).

Mutation of *vang-1* did not restore posterior QL.pax localization in *bar-1*/β-catenin and *pop-1*/TCF mutants, indicating that *vang-1* acts upstream of *bar-1*/β-catenin (S2A Fig). This is in agreement with the observation that *mab-5* expression is lost in the QL lineage of *sfrp-1*; *vang-1 bar-1* triple mutants (S3 Fig). Loss of *vang-1* also had no significant effect on QL.pax localization in *egl-20* mutants or mutants of the Wnt secretion factor *mig-14*/Wntless [38] (Fig 4B), indicating that loss of *vang-1* does not activate canonical Wnt signaling in the absence of Wnt ligand stimulation.

### VANG-1 overexpression inhibits canonical Wnt/β-catenin signaling in the QL lineage

**Fig 5:**
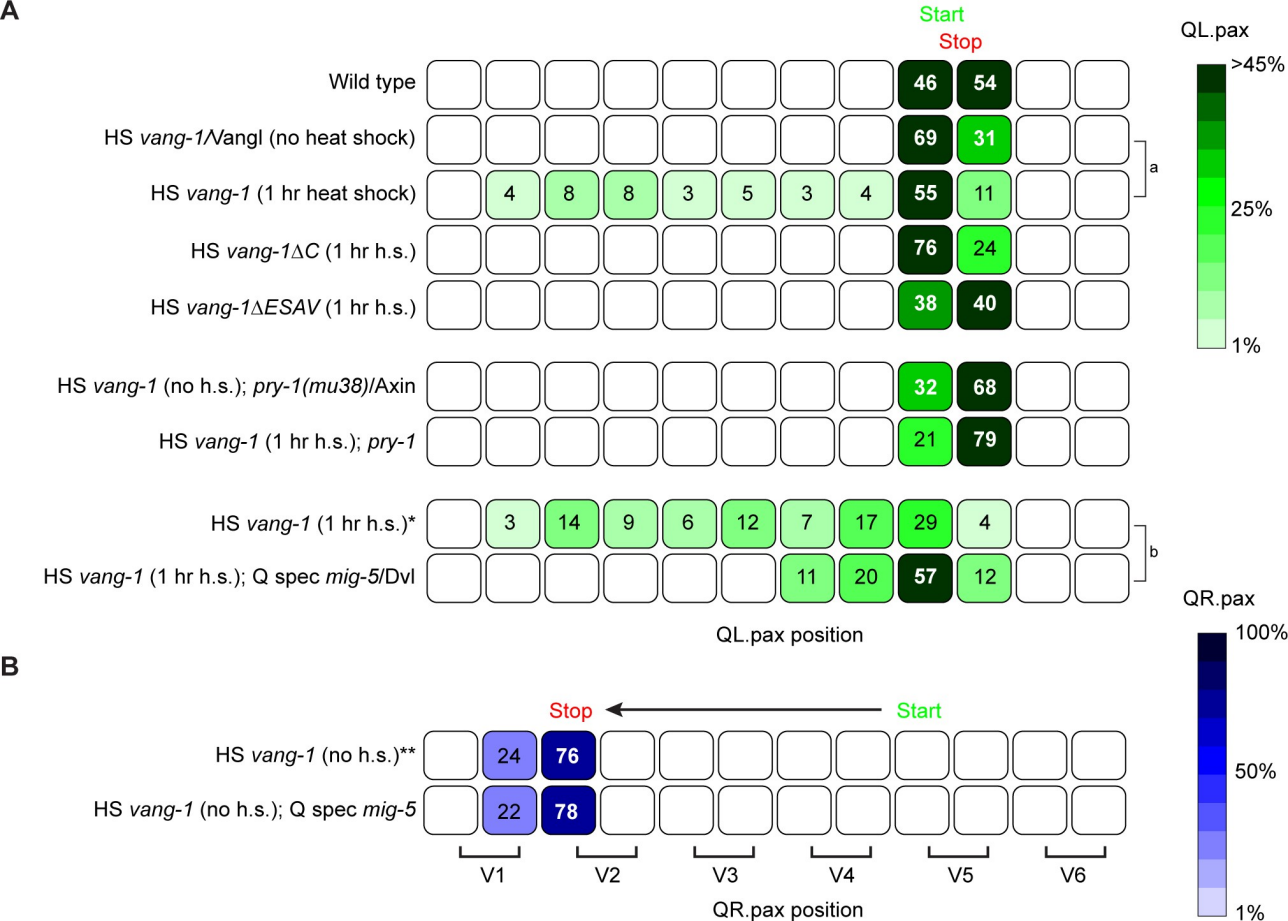
VANG-1/Vangl negatively regulates canonical Wnt/β-catenin signaling. Final positions of the QL.pax (**A**) and QR.pax (**B**) with respect to the seam cells V1.a to V6.p (lower brackets indicate Vn.a (left) and Vn.p (right) daughters of Vn cells). Values listed are the cumulative percentiles of the total number of cells scored in at least 3 independent experiments, n>30 for each experiment. The *hsp16* promoter was used for inducible overexpression of wild type and ΔC *vang-1* (HS *vang-1* and HS *vang-1Δ*C) and the *lin-32* promoter was used to specifically express *mig-5* in the Q lineage (Q spec *mig-5*) [54]. Statistical significance was calculated using a Student’s t-test (a, b p<0.05). *, ** siblings of the HS (*Phsp16*) *vang-1*; Q spec (*Plin-32*) *mig-5* strain that have lost the extrachromosomal *mig-5* transgene. h.s., heat shock.

To examine the effect of increased VANG-1 activity, we used a heat shock promoter (HS *vang-1*) to drive expression of VANG-1 at the beginning of Q neuroblast development. We found that overexpression of VANG-1 induced significant anterior migration of the QL descendants (Fig 5A). This phenotype is opposite to loss of *vang-1*, and is in agreement with a negative regulatory role of VANG-1 in BAR-1/β-catenin dependent Wnt signaling.

To further refine the epistatic relationship between *vang-1* and the canonical Wnt pathway, we overexpressed VANG-1 in a *pry-1* mutant background. *pry-1* encodes an ortholog of Axin, an essential component of the β-catenin destruction complex that negatively regulates BAR-1/β-catenin signaling in the Q neuroblast lineage [26]. We found that in this background, overexpression of VANG-1 had no effect on QL.pax positioning (Fig 5A). Together with the double mutant analysis discussed above, these results demonstrate that VANG-1 acts at a level between Frizzled and the BAR-1/β-catenin destruction complex.

### VANG-1/Vangl is an evolutionarily conserved negative regulator of canonical Wnt/β-catenin signaling

**Fig 6:**
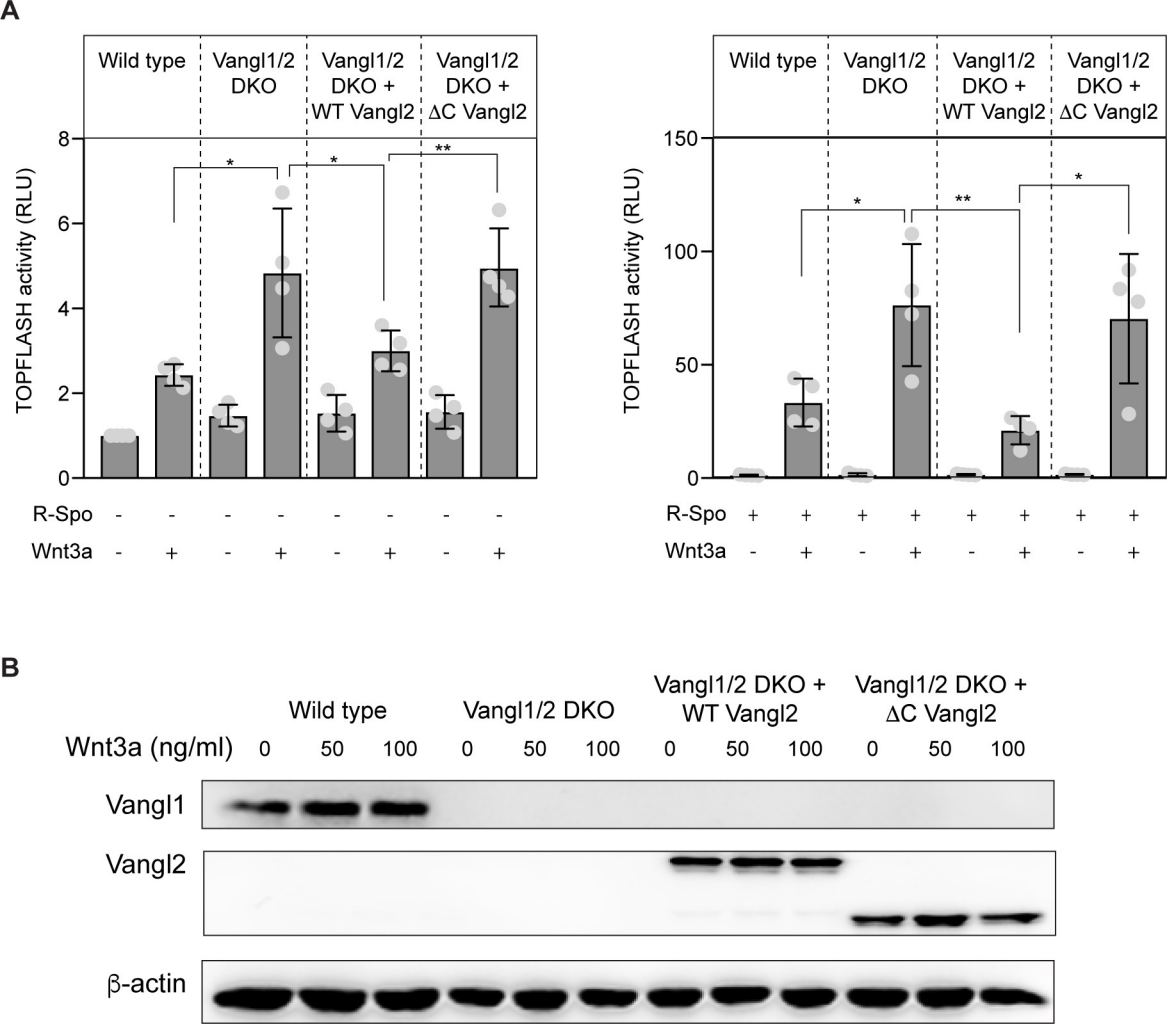
Vangl negatively regulates canonical Wnt/β-catenin signaling in mammalian cells. (**A**) TOPFLASH reporter activity in wild type and Vangl1/2 double mutant (DKO) HEK293 cells treated with 100 ng/ml Wnt3a or the corresponding amount of 0.1% BSA in PBS in the absence (left panel) or presence (right panel) of R-Spondin. Rescue experiments were performed with expression of wild type and C-terminally truncated Vangl2 (fused to EGFP). Statistical significance was calculated using a Student’s t-test. *, p<0.05; **, p<0.01. (**B**) Western blot analysis of Vangl1 and Vangl2 expression in control, Vangl1/2 DKO and DKO cells expressing wild type or C-terminally truncated Vangl2 (fused to EGFP). Note that endogenous Vangl2 expression is not visible at this exposure. A longer exposure is provided in S4C Fig.

It has previously been shown that overexpression of Vangl in mammalian tissue-culture cells inhibits the activity of the β-catenin-responsive reporter TOPFLASH in a dose-dependent manner [11]. Moreover, using morpholino mediated knock down, a reduction in Vangl was associated with increased TOPFLASH activity [11] and Wnt/β-catenin signaling related phenotypes during zebrafish development [13, 14]. To further investigate the role of Vangl as a negative regulator of Wnt/β-catenin signaling in mammalian cells, we used CRISPR/Cas9 mediated genome editing to mutate Vangl1 and its paralog Vangl2 in HEK293 cells. We targeted the fourth exon of Vangl1 and the third exon of Vangl2, and isolated a clone in which Vangl1 contains a 1 nucleotide deletion and Vangl2 a 1 nucleotide insertion (S4A Fig). Both mutations induce a shift in reading frame and are predicted to disrupt gene function. Consistently, no Vangl1 or Vangl2 protein could be detected in the double mutant cells (S4B Fig). Similar to loss of *vang-1* in *C*. *elegans*, we found that the combined loss of Vangl1 and Vangl2 strongly enhanced canonical Wnt signaling. As shown in Fig 6A, there was a significant increase in Wnt3a induced TOPFLASH activity. An even more robust increase was observed when the cells were also exposed to R-Spondin, which facilitates canonical Wnt signaling through an Lgr5 and RNF43 dependent mechanism [39]. In both cases, the increased response to Wnt3a was fully rescued by expression of wild type Vangl2. Moreover, as we observed for *vang-1* in *C*. *elegans*, loss of Vangl1 and Vangl2 was not sufficient to induce Wnt reporter activity in the absence of Wnt ligand stimulation. These results are in agreement with the morpholino based studies in zebrafish, and show that VANG-1/Vangl has an evolutionarily conserved function as a negative regulator of canonical Wnt/β-catenin signaling.

### The carboxy-terminal PDZ-binding motif of VANG-1/Vangl is required for inhibition of canonical Wnt/β-catenin signaling

The inhibitory effect of Vangl overexpression in mammalian cells was found to be dependent on the carboxy-terminal PDZ binding (PBM) motif of Vangl [11]. Consistently, we found that the enhanced response to Wnt3a in Vangl1/2 double mutant cells, which was rescued by expression of wild type Vangl2, was not reverted by expression of a carboxy-terminally truncated mutant of Vangl2 that lacks this PDZ binding motif (Fig 6A).

We found that the carboxy-terminal PBM was also essential for the inhibitory effect of *C*. *elegans* VANG-1. Thus, deletion of the carboxy-terminus (*vang-1ΔC*) or the four terminal amino acids that constitute the VANG-1 PBM (*vang-1ΔESAV*) [22] fully disrupted the inhibitory effect of VANG-1 overexpression on posterior QL.pax positioning (Fig 5A), demonstrating the evolutionarily conserved role of the PDZ binding motif in cross-talk between VANG-1/Vangl and canonical Wnt/β-catenin signaling.

### VANG-1 inhibits clustering of MIG-5/Dvl at the plasma membrane

Previous studies have shown that the carboxy-terminus of Vangl binds to the PDZ domain of Dvl [11, 40]. In *C*. *elegans*, VANG-1 binds to two of the three Dvl orthologs, DSH-2 and MIG-5, through an interaction that is dependent on the PDZ binding motif [22]. The PBM is also required for binding of the junctional protein DLG-1 (which is not expressed in the migrating Q neuroblasts, S5 Fig), but does not constitute a general PDZ binding site, as VANG-1 does not bind to DSH-1/Dvl or other PDZ domain containing proteins such as Bazooka [22].

**Fig 7:**
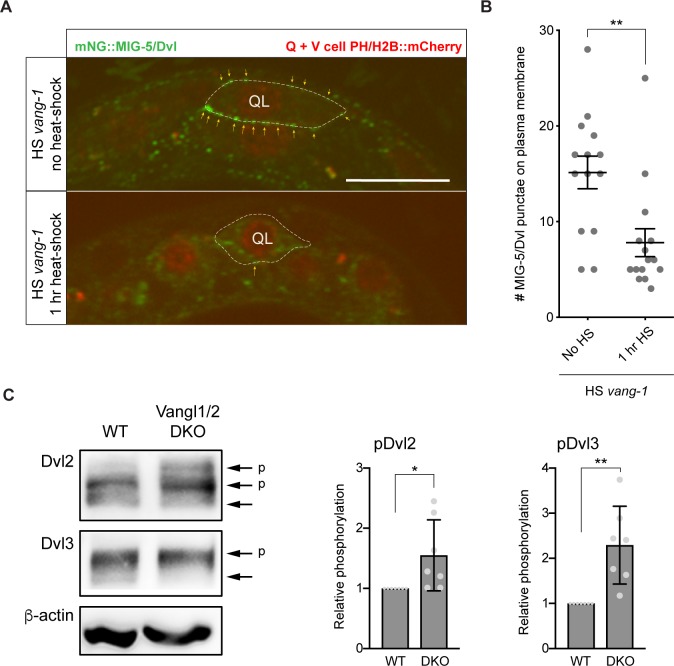
VANG-1/Vangl controls Dvl localization and phosphorylation. (**A**) Expression of endogenously tagged mNG::MIG-5/Dvl [41] in the QL neuroblast (outlined with dotted line). Punctae of mNG::MIG-5 at the cell membrane are indicated with yellow arrows. *wrt-2* promoter directed expression of PH::mCherry and H2B::mCherry was used to mark the Q neuroblasts and seam cells. VANG-1 was overexpressed using the *hsp16* heat-shock (HS) promoter (1 hr. heat-shock). Scale bar = 10 μm. (**B**) Quantification of MIG-5/Dvl punctae on the Q neuroblast plasma membrane. Statistical significance was calculated using a Student’s t-test. **, p<0.01. (**C**) Loss of Vangl1/2 increases Dvl2 and Dvl3 phosphorylation. Endogenous Dvl2 and Dvl3 were detected using specific antibodies. Phosphorylation of Dvl2 and Dvl3 (indicated by arrows with a p) was quantified in wild type and Vangl1/2 double mutant (DKO) cells. Statistical significance was calculated using a Student’s t-test.*, p<0.05; **, p<0.01.

Of the three Dvl orthologs, only MIG-5 is required for Wnt/β-catenin signaling in the Q neuroblast lineage [26]. Knock-in of mNeonGreen (mNG) at the amino-terminus of MIG-5/Dvl showed that it is strongly expressed in the QL and QR neuroblasts and their descendants [41], confirming previous results with reporter transgenes [42]. We observed that endogenous mNG::MIG-5/Dvl forms distinct punctae on the plasma membrane of QL and QR (Fig 7A). This punctate localization was strongly reduced when VANG-1 was overexpressed (Fig 7A and 7B), indicating that VANG-1 inhibits clustering of MIG-5/Dvl at the plasma membrane.

Dvl is a highly versatile protein that functions in both canonical and non-canonical Wnt signaling [43]. How the canonical and non-canonical signaling activity of Dvl is regulated and separated is still poorly understood. We propose that by binding MIG-5/Dvl and inhibiting its clustering at the plasma membrane, VANG-1 may restrict the pool of MIG-5 that is available for canonical Wnt signaling. Consistently, we found that a mild increase in MIG-5/Dvl expression (Q spec *mig-5*)–below the threshold for activation of canonical Wnt signaling (Fig 5B)–was sufficient to abrogate the inhibitory effect of VANG-1 overexpression (Fig 5A). Importantly, we also found that in mammalian Vangl1/2 double mutant cells, there is a significant increase in phosphorylation of Dvl2 and Dvl3 (Fig 7C). Increased phosphorylation is a hallmark of Dvl activity in both canonical and non-canonical Wnt signaling [44]. However, given the enhanced TOPFLASH reporter activity in the Vangl1/2 double mutant cells, this increase in phosphorylation most likely reflects Dvl activity in the canonical Wnt pathway. This is in agreement with our model that in the absence of Vangl, more Dvl is available for signaling through the Wnt/β-catenin pathway.

### *prkl-1*/Pk does not modulate canonical Wnt/β-catenin signaling in the Q neuroblast lineage

In contrast to *vang-1*, loss of *prkl-1*/Pk did not induce posterior displacement of the QR descendants in the *sfrp-1* mutant background (S2C Fig). Furthermore, *prkl-1* did not influence the sensitivity of QL and QR to ectopically expressed *egl-20* (Fig 3) nor did it suppress the Wnt signaling defect of *mig-1* and *lin-17* Frizzled mutants (S2B Fig). Despite having no apparent role in canonical Wnt signaling, smFISH analysis revealed that *prkl-1* is expressed in the Q neuroblasts (S1 Fig). With a gradual decrease in transcript number during the initial, Wnt independent polarization and short-range migration of QL and QR, the expression dynamics of *prkl-1* are however opposite to *vang-1*. We conclude that *vang-1* functions independently of *prkl-1* in modulating Wnt/β-catenin signaling in *C*. *elegans*.

## Discussion

Vangl and Pk are core components of the planar cell polarity pathway [4]. In addition to their role in epithelial polarity and cell migration, studies in vertebrate model systems have suggested that there may be an antagonistic relationship between Vangl2 and Pk and the canonical Wnt/β-catenin pathway [11–15]. To study this potential cross-talk, we performed a comprehensive mutant analysis in *C*. *elegans*. We show that the Vangl ortholog VANG-1 negatively regulates canonical Wnt signaling in migrating Q neuroblasts. This inhibitory activity depends on the carboxy-terminal PDZ binding motif of VANG-1 and the Dvl ortholog MIG-5, but is independent of the Pk ortholog PRKL-1. Moreover, using mammalian Vangl1/2 double mutant cells, we show that loss of Vangl enhances Wnt3a signaling and induces hyperphosphorylation of Dvl. We conclude that Vangl has an evolutionarily conserved function in dampening canonical Wnt signaling.

The Q neuroblasts QL and QR have different response thresholds for canonical Wnt pathway activation [25]. At physiological levels of the Wnt ligand EGL-20, QL activates canonical Wnt signaling and expression of the target gene *mab-5*/Hox, while QR adopts a non-canonical Wnt signaling response that enables the QR descendants to migrate anteriorly [32]. We found that this response threshold is lowered in *vang-1* mutants, leading to ectopic canonical Wnt pathway activation and *mab-5* expression in QR when EGL-20/Wnt signaling activity is increased. Loss of *vang-1* also lowers the threshold for canonical Wnt signaling in QL, indicating that *vang-1* is not responsible for the difference in signaling response between the two Q neuroblasts. Consistently, smFISH analysis showed that *vang-1* displays similar expression dynamics and transcript numbers in QL and QR.

Even though loss of *vang-1* reduces the threshold for canonical Wnt signaling, we did not observe ectopic activation of canonical Wnt signaling at physiological levels of the EGL-20/Wnt ligand. This indicates that VANG-1 primarily functions as a buffer that prevents erroneous canonical Wnt pathway activation at elevated Wnt ligand levels. Our observation that loss of Vangl1/2 increases the response to Wnt3a, but does not induce activation of canonical Wnt signaling on its own, indicates that Vangl has a similar function in vertebrates. This is also in agreement with studies in zebrafish, where mutation of Vangl2 does not induce ectopic canonical Wnt pathway activation [5], but enhances the response to ectopic expression of the canonical Wnt ligand Wnt8a [13].

Studies in zebrafish and mouse have shown that inhibition of canonical Wnt signaling depends on the interaction of Vangl2 with the cytoplasmic signaling protein RACK1 [14] or the atypical Wnt receptor Ror2 [15]. Our results indicate that VANG-1 acts through a different mechanism in *C*. *elegans*. We found that knock down of the RACK1 ortholog *rack-1* inhibits the EGL-20/Wnt dependent posterior migration of the QL descendants [45], demonstrating that it functions as a positive rather than a negative regulator of canonical Wnt signaling in this system. The Ror2 ortholog CAM-1 is required for the persistent polarization and migration of the QR descendants [32]. In contrast to *vang-1*, we observed no posterior shift in QR.pax position in *cam-1*; *sfrp-1* double mutants and no effect of adding a mutation in the Wnt target gene *mab-5*/Hox (S2D Fig), indicating that *cam-1*/Ror2 does not influence canonical Wnt signaling in the Q neuroblast lineage.

Our results show that in the Q neuroblast lineage, cross-talk between VANG-1 and the canonical Wnt pathway is mediated at the level of the cytoplasmic signaling protein Dvl. We found that *vang-1* acts genetically upstream of the Dvl ortholog *mig-5* and that deletion of a conserved, carboxy-terminal Dvl interaction domain eliminates the inhibitory effect of VANG-1 overexpression. Moreover, we found that overexpression of VANG-1 strongly decreases clustering of endogenous MIG-5/Dvl at the plasma membrane, and that mild overexpression of MIG-5 –at a level below the threshold for activation of canonical Wnt signaling–was sufficient to abrogate the inhibitory effect of VANG-1 on canonical Wnt signaling. Based on these results, we propose that VANG-1 dampens canonical Wnt signaling by sequestering MIG-5/Dvl and thereby restricting the pool of MIG-5/Dvl that is available for signaling. The increased phosphorylation of Dvl that we observed in mammalian Vangl1/2 double mutant cells, which together with the increase in TOPFLASH reporter activity is indicative of Dvl activity in canonical Wnt signaling [44], is consistent with such a model.

Studies in vertebrates suggest that Pk may also negatively regulate canonical Wnt signaling. Pk has been shown to bind and destabilize Dvl, possibly by promoting its degradation [46, 47]. Furthermore, overexpression of Pk was found to inhibit canonical Wnt signaling in reporter gene assays [12, 47]. However, our genetic analysis did not reveal a role for *prkl-1* in canonical Wnt signaling. Even though *prkl-1* is expressed in the Q neuroblasts [32], loss of *prkl-1* did not influence the response to EGL-20/Wnt. Our results therefore support the conclusion that PRKL-1 is not part of the cross-talk between VANG-1 and the canonical Wnt pathway.

Deregulation of canonical Wnt signaling is one of the primary causes of colon cancer [1]. Recent clinical studies have demonstrated that Vangl2 is frequently silenced by promoter hypermethylation in colon cancer cell lines and tumor samples [48]. Our results on the inhibitory effect of VANG-1/Vangl on canonical Wnt signaling provide a mechanistic explanation for the role of Vangl2 as a tumor suppressor gene.

## Materials and methods

### *C*. *elegans* strains and culture

Unless noted otherwise, *C*. *elegans* strains were cultured at 20°C using standard conditions. As wild type, the Bristol N2 strain was used. Mutant alleles and transgenes used in this study are LGI: *mig-1(e1787)*, *lin-17(n671)*, *pop-1(hu9)*, *pry-1(mu38)*; LGII: *cam-1(gm122)*, *mig-14(mu71)*, *mig-5(cp385[mNG-GLO^AID*::*mig-5)* [41]; LGIII: *mab-5(gk670)*; LGIV: *unc-5(e53)*, *sfrp-1(gk554)*, *prkl-1(ok3182)*, *egl-20(n585)*, *dpy-20(e1362)*; LGV: *heIs63 [Pwrt-2*::*gfp-ph; Pwrt-2*::*h2b-gfp; Plin-48*::*mTomato]* [49], *muIs53 [Phsp16*::*egl-20; unc-22(dn)]* [25]; LGX: *vang-1(tm1422)*, *bar-1(ga80)*, *dlg-1(cp30[dlg-1*::*mNG-C1^3xFlag)* [41] and unassigned, *huIs143 [Phsp16*::*vang-1; Pmyo-2*::*mTomato]* and the extra-chromosomal transgenes *huEx279*, *huEx280*, *huEx281 [Pegl-17*::*vang-1-gfp; Pmyo-2*::*mTomato]*, *huEx654*, *huEx655 [Phsp16*::*vang-1ΔC; Phsp16*::*gfp; Plin-48*::*mTomato]*, *huEx673 [Plin-32*::*mig-5*::*gfp; Plin-48*::*mTomato]*, *huEx754 [Phsp16*::*vang-1ΔESAV; Pmyo-2*::*gfp]*. To construct the *sfrp-1(gk554) prkl-1(ok3182)* double mutant, first an *unc-5(e53) prkl-1(ok3182) dpy-20(e1362)* triple mutant was constructed. Triple mutants were crossed to wild type males and the resulting heterozygous males were in turn crossed to *sfrp-1(gk554)* hermaphrodites. The F2 generation was screened for Dpy non-Unc animals homozygous for *prkl-1(ok3182)* and heterozygous for *sfrp-1(gk554)*. The *dpy-20(e1362)* allele was subsequently removed by outcrossing.

### Molecular biology

Total RNA was isolated from mixed stage animals and reversed transcribed to obtain cDNA. Individual cDNA clones encoding the *vang-1a* isoform and *mig-5* were PCR amplified from mixed stage cDNA using primers containing attB1 and attB2 Gateway recombination sites. cDNA fragments were subsequently recombined with pDNR221 to generate Gateway compatible entry clones. Promoter sequences of *egl-17* and *lin-32* (4.6 kb or 3.0 kb of upstream sequence, respectively) were cloned by PCR amplifying from genomic DNA using primers containing attB4 and attB1R Gateway recombination sites and recombined with pDNRP4-P1R to generate entry clones. Expression constructs were generated by combining appropriate entry clones in the pCFJ150 expression vector. Expression constructs were injected at 20–50 ng/μl with 5–10 ng/μl co-injection marker and supplemented with pBluescriptII to 150 ng/μl. The *Phsp16*::*vang-1* construct was made by PCR amplifying *vang-1a* from the Gateway donor vector and cloning the PCR product into the pPD49.87 heat shock vector using NheI and BamHI restriction sites. The *Phsp16*::*vang-1ΔC* construct was made by deleting the carboxy-terminal intracellular domain of *vang-1a* [11] by using primers 5’-GGGGACAAGTTTGTACAAAAAAGCAGGCTatgtcgtatcaagataacag-3’ and 5’-GGGG ACCACTTTGTACAAGAAAGCTGGGTtcactcgaggagcacaa-cagaga-3’. *Phsp16*::*vang-1ΔESAV* was made by deleting the codons for the last four amino acids of *vang-1a*, using primers 5’-GGGGACAAGTTTGTACAAAAAAGCAGGCTTTatgctgtatcaagataacag-3’ and 5’-GGGGACCACTTTGTACAAGAAAAGCTGGGTTtcaatt-gctaatttttaagg-3’.

### Phenotypic analysis and microscopy

The positions of the final QL and QR cell descendants were scored with DIC microscopy in late L1 stage larvae as previously described [35]. Animals were mounted on 2% agarose pads containing 10 mM sodium azide for immobilization.

### Time lapse live cell imaging

To follow Q cell migration in time, synchronized animals (4–5 hours after hatching) carrying the *heIs63[Pwrt-2*::*gfp-ph; Pwrt-2*::*h2b-gfp; Plin-48*::*mTomato]* transgene were mounted in 0.5 μl of 0.1 μm diameter polystyrene beads in aqueous solution (Polysciences 00876 2.5% w/v aqueous suspension) on 10% agarose pads [50]. Images were acquired using a Leica TCS SPE confocal microscope (63x objective, zoom 1.0x, 10% 488 nm laser power). Z-stacks of 0.5 μm per stack were made every 15 minutes for 7 hours. Images were acquired using LASAF software. Image processing and analysis were performed using ImageJ v1.43u and movie construction using Adobe Photoshop CS5.1 software.

### Heat shock experiments

Heat shock experiments on animals carrying the *muIs53 [Phs*::*egl-20; unc-22(dn)]* transgene were performed as previously described [35], except as follows: synchronized L1 larvae (aged 0–1 hours after hatching) were incubated at 33°C in a volume of 50 μl M9 buffer for 2, 3 or 4 minutes. Placing the tubes on ice for 10 seconds ended the heat shock treatment. Animals were cultured overnight on OP50 seeded NGM agar plates at 20°C and the position of the Q cell descendants was scored the following morning. Heat shock experiments on animals carrying the *huIs143 [Phsp16*::*vang-1; Pmyo-2*::*mTomato]*, *huEx654*, *huEx655 [Phsp16*::*vang-1ΔC; Phsp16*::*gfp; Plin-48*::*mTomato]* or *huEx754 [Phsp16*::*vang-1ΔESAV; Pmyo-2*::*gfp]* were performed similarly, except that the heat shock treatment was for one hour and tubes were not chilled on ice.

### Single molecule fluorescence in situ hybridization

The smFISH protocol was performed as described elsewhere [34]. In brief, synchronized L1 larvae were fixed using 4% paraformaldehyde and suspended in 70% ethanol. Hybridization was done overnight at 33°C in the dark. Short oligonucleotide probes were designed using a web-based algorithm (www.singlemoleculefish.com) and chemically coupled to the fluorescent dye Cy5. Animals were suspended in buffer containing DAPI for nuclear counterstaining before mounting. Z-stacks with a slice thickness of 0.5 μm were obtained with a Leica DM6000 microscope, equipped with a Leica DFC360FX camera, 100x oil objective and Y5 (Cy5), A4 (DAPI) and GFP filter cubes. Images were acquired with 1024x1024 resolution and subjected to a 2x2 binning. Quantification of mRNA was performed manually on the obtained z-stacks. Only mRNA spots visible in at least two independent focal planes were counted. The Q cell boundary was marked by the *heIs63* transgene. Analysis was performed using ImageJ v1.43u software.

### Statistical analysis of Q neuroblast migration

The final position of the Q descendants was used as an assay to determine canonical Wnt/β-catenin pathway activity: QL.pax cells were scored as having activated canonical Wnt signaling if located on V4.p or more posterior, QR.pax cells if located on V3.p or more posterior. Each strain was scored in 3 independent experiments. Statistical comparisons were performed using unpaired, two tailed Student’s t-tests.

### Mammalian cell culture and treatments

T-REx-293 cells (Thermo Fisher Scientific) were propagated in Dulbecco's modified Eagle's medium (DMEM, 41966–029, Gibco, Life Technologies) supplemented with 10% fetal bovine serum (FBS, 10270–106, Gibco, Life Technologies), 2 mM L-glutamine (25030024, Life Technologies), 1% penicillin-streptomycin (XC-A4122/100, Biosera). Treatments used were: recombinant human Wnt3a (CF 5036-WN-CF RnD Systems), recombinant human R-Spondin-1 (120–38, PeproTech) and LGK 974 (974–02, Stem RD) for inhibition of endogenous Wnt ligands secretion.

### CRISPR/Cas9 mediated targeting of Vangl1 and Vangl2

Sequences of guide RNAs targeting human Vangl1 (GCATTTTGGACTCTCGGGAC) and Vangl2 (GGACAATGAGTCCACACGAG) were inserted into plasmids encoding Cas9 and a fluorescent reporter according to the cloning protocol described by the manufacturer (pU6-(BbsI)_CBh-Cas9-T2A-mCherry, Addgene #64324 for Vangl1, and pSpCas9(BB)-2A-GFP (PX458), Addgene #48138, for Vangl2). Plasmids were transiently transfected using Lipofectamine 2000 (Invitrogen) into T-REx 293 cells. After 24 hr, cells were trypsinized, FACS sorted, and GFP and mCherry double positive cells were plated as single cells in 96-well plates. After an expansion time of approximately 2–3 weeks, cells were trypsinized. Part of the cellular suspension was taken for genomic DNA (gDNA) isolation, while the rest of the cells was further expanded. gDNA isolation was performed using DirectPCR Lysis Reagent (Cell) (Viagen Biotech), Proteinase K (EO0491, Thermo Fisher Scientific) and DreamTaq DNA Polymerase (EP0701, Thermo Fisher Scientific) according to the manufacturer’s recommendations. Isolated gDNA was used for amplification of a region surrounding the place of Cas9 cutting. The resulting PCR product was subsequently digested with BsoBI (R0586, NEB) for Vangl1 and BssSI (R0587, NEB) for Vangl2 to detect disruption of gRNA targeted restriction sites. The positive clones were selected for further screening by whole-cell lysate western blot analysis. Final screening of clones was performed by sequence analysis using the Illumina platform and compared with the reference sequence [51].

### Transfection

Cells were seeded in 24-well plates 24 hours prior to transfection with 1 μg/ml, pH 7.4 polyethylenimine (PEI) and plasmid DNA, at a ratio of 4 μl PEI to 1 μg DNA as described [52]. Briefly, for transfection of 1 well of 24-well plate 0,4 μg of total plasmid DNA was used. For dual luciferase assays, 0.1 μg of the pRLtkLuc plasmid and 0.1 μg of the Super8X TopFlash were co-transfected. For rescue experiment in Vangl1/2 DKO cells, 0.01 μg of wild type or ΔC Vangl2 plasmids [53] were used. After 6 hr, the culture medium was changed, treated as indicated and cultured for 16 hours before further analysis.

### Dual luciferase reporter and disheveled shift assays

For analysis of canonical Wnt signaling activity, transfected cells were treated overnight with 0,1 μM LGK-974 (all samples), 50 ng/ml of recombinant R-Spondin-1, 50 and 100 ng/ml of recombinant Wnt3a or 0,1% BSA in PBS as indicated. Dual luciferase assays were performed according to the protocol provided by the manufacturer (E1960; Promega) and luminescence was analyzed using a Hidex Bioscan Plate Chameleon Luminometer. Results are presented as ratios of firefly to renilla measurements and normalized to the unstimulated control condition. Results were analyzed by unpaired t-test, with >3 replicate experiments. For analysis of Dvl2 and Dvl3 phosphorylation, cells were lysed and analyzed by Western Blot.

### Western blot analysis

To examine Dvl2 and Dvl3 phosphorylation, culture medium was removed and lysis was performed directly on the plate in 100 mM Tris HCl pH 6.8, 20% glycerol, 1% SDS, 0.01% bromophenol blue and 1% 2-mercaptoethanol. For comparison of parental and Vangl1/2 DKO lines, cells were detached, washed in PBS, lysed in 1% SDS, 10mM EDTA and 50mM Tris, pH 6.8, protein concentration was measured by DC Protein Assay (5000111, Bio-Rad) and samples containing 20 μg of protein were analyzed. Western blotting was performed according to the manufacturer’s instructions, in short samples were sonicated and boiled for 5 min, loaded on 8% SDS-PAGE gel, electrophoresis was performed on 120 V, proteins were transferred on PVDF membrane for 1h 15 min on 100 V. Then membranes were blocked in 5% non-fat dry milk solution in TBS-T for 30 min and subsequently incubated overnight with primary antibodies in blocking buffer: DVL2 (1:1000, Cell Signaling Technology, cs3216), Dvl3 (1:1000, Cell Signaling Technology, cs3218), β-actin (1:3000, Cell Signaling Technology, cs4970), Vangl1 (1:500, Santa Cruz Biotechnology, sc-166844) and Vangl2 (1:1000, Merck, MABN750). Next, membranes were washed in TBS-T and incubated with HRP-conjugated secondary antibodies, washed and chemiluminescence was recorded using a Fusion SL imaging system (Vibler) using Immobilon Western Chemiluminescent HRP Substrate (Merck, WBKLS0500). Results were quantified by densitometric analysis of western blot bands using Fiji distribution of ImageJ and analyzed by t-test. Disheveled phosphorylation status is represented as the ratio of the peak area for the upper band representing P-Dvl to the peak area of the lower band (Dvl).

## Supporting information

S1 Fig *vang-1*/Vangl and *prkl-1*/Pk are expressed in both the QL and QR lineage and show opposite expression dynamics during initial polarization and migration. Transcription dynamics of *vang-1* (left panels) and *prkl-1* (right panels) as quantified in QL (grey), QL.p (red), QR (blue) and QR.p (green), n>35 for both genes. The number of mRNA spots per cell is plotted against the cell position with respect to the seam cells V4 and V5. The direction of the initial polarization and migration of QL and QR is indicated by an arrow.
(TIF)

S2 Fig *vang-1*/Vangl acts upstream of *bar-1*/β-catenin and *pop-1*/TCF, and loss of *prkl-1*/Pk and *cam-1*/Ror do not affect canonical Wnt signaling. (**A**-**D**) Final positions of the QL.pax (**A**, **B**) and the QR.pax (**C**, **D**) with respect to the seam cells V1.a to V6.p (lower brackets indicate Vn.a (left) and Vn.p (right) daughters of Vn cells). Values listed are the cumulative percentiles of the total number of cells scored in at least 3 independent experiments, n>30 for each experiment. Statistical significance was calculated using a Student’s t-test (a, p<0.05).
(TIF)

S3 Fig Expression of *mab-5*/Hox mRNA is lost in the QL lineage in *sfrp-1*/SFRP; *vang-1*/Vangl; *bar-1*/β-catenin triple mutants. Displayed are the *mab-5* mRNA counts in QL (after completion of initial migration) and QL.p.
(TIF)

S4 Fig Generation of Vangl1 and Vangl2 double mutant HEK293 cells. (**A**) Sequences of the guide RNAs and the mutations in the double mutant (DKO) cell line. (**B**) Western blot analysis of Vangl1 and Vangl2 expression in wild type and Vangl1/2 DKO cells. (**C**) Western blot analysis of Vangl1 and Vangl2 expression in control, Vangl1/2 DKO and DKO cells expressing wild type or C-terminally truncated Vangl2 (fused to EGFP). A longer exposure of Vangl2 expression is shown.
(TIF)

S5 Fig Expression of endogenous *dlg-1::mNG* [41]. DLG-1 is expressed in the seam (V) cells, but no expression is visible in the QL neuroblast (position marked by arrow). Scale bar = 10 μm.
(TIF)

S1 Movie Live time-lapse confocal imaging of wild type animals expressing plasma membrane and nuclear localized GFP in QR, QR.a (anterior daughter cell), QR.p (posterior daughter cell) and the seam (V) cells (transgene *heIs63*). Anterior is left. Images were acquired every 15 minutes for a total duration of 7 hours. Movies are played at 2 frames per second.
(AVI)

S2 Movie Live confocal imaging of *sfrp-1(gk554); vang-1(tm1422)* double mutants. Conditions as in S1 Movie. Note that QR.p stays in the posterior.
(AVI)

S1 Dataset Numerical data of graphs presented in main and supplementary figures. (XLSX)
