# Supplementary figures and images for: The planar cell polarity protein VANG-1/Vangl negatively regulates Wnt/β-catenin signaling through a Dvl dependent mechanism
Source: PLoS Genet. 2018 Dec 7;14(12):e1007840. doi: 10.1371/journal.pgen.1007840 (PMC6307821; doi:10.1371/journal.pgen.1007840)

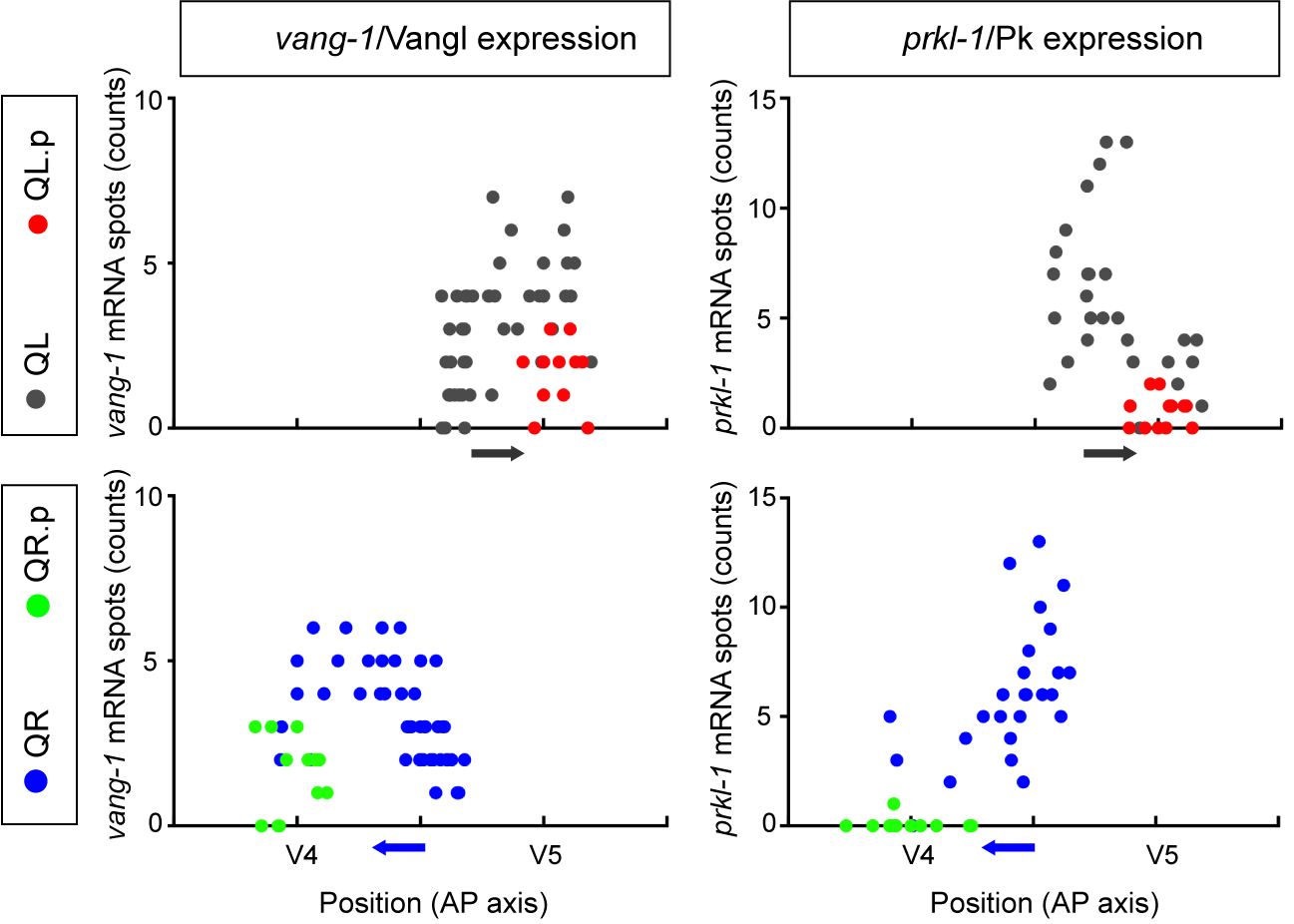

Supplement: S1 Fig — Transcription dynamics of vang-1 (left panels) and prkl-1 (right panels) as quantified in QL (grey), QL.p (red), QR (blue) and QR.p (green), n>35 for both genes. The number of mRNA spots per cell is plotted against the cell position with respect to the seam cells V4 and V5. The direction of the initial polarization and migration of QL and QR is indicated by an arrow. (TIF) [file pgen.1007840.s001.tif]

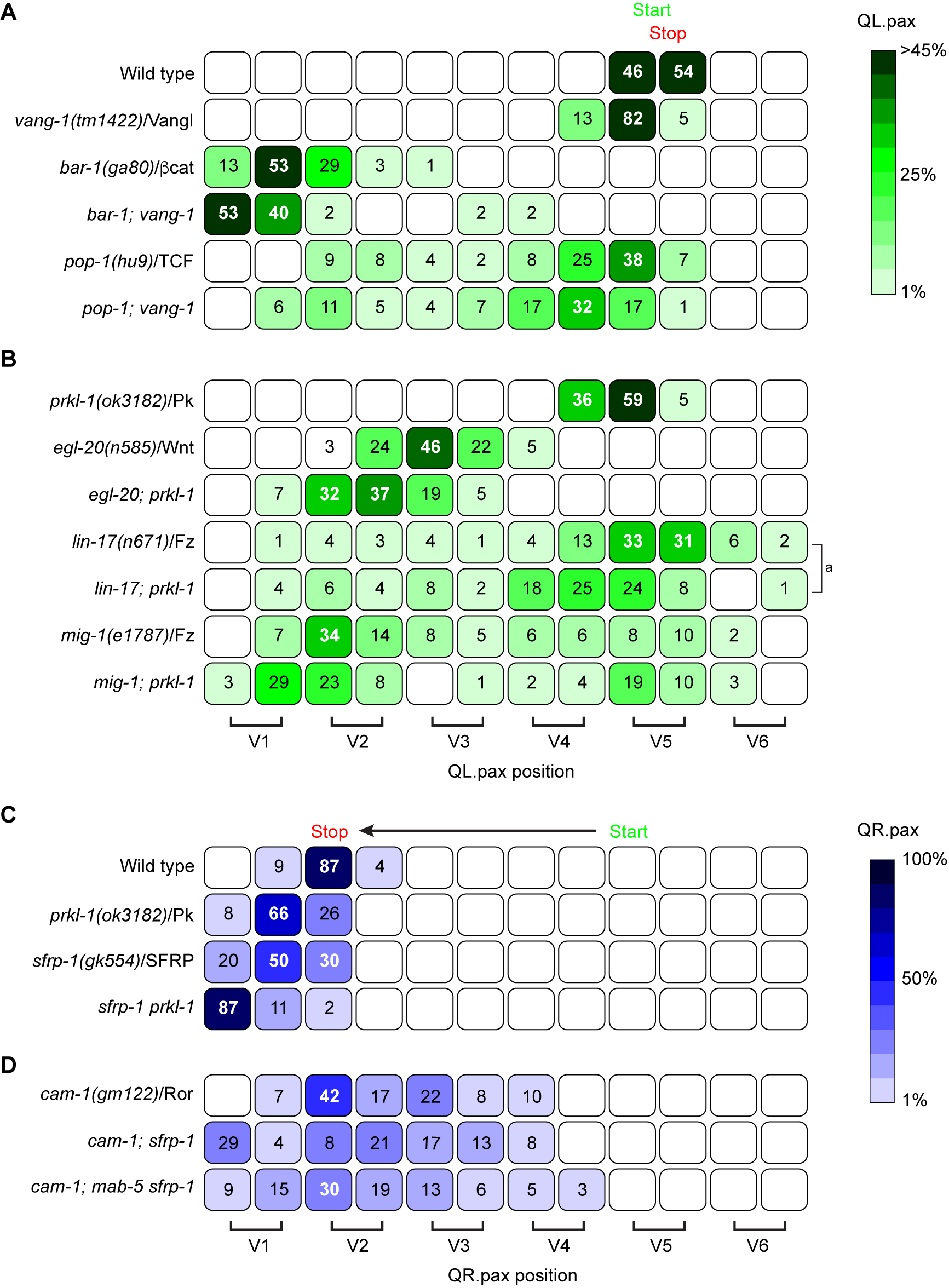

Supplement: S2 Fig — (A-D) Final positions of the QL.pax (A, B) and the QR.pax (C, D) with respect to the seam cells V1.a to V6.p (lower brackets indicate Vn.a (left) and Vn.p (right) daughters of Vn cells). Values listed are the cumulative percentiles of the total number of cells scored in at least 3 independent experiments, n>30 for each experiment. Statistical significance was calculated using a Student’s t-test (a, p<0.05). (TIF) [file pgen.1007840.s002.tif]

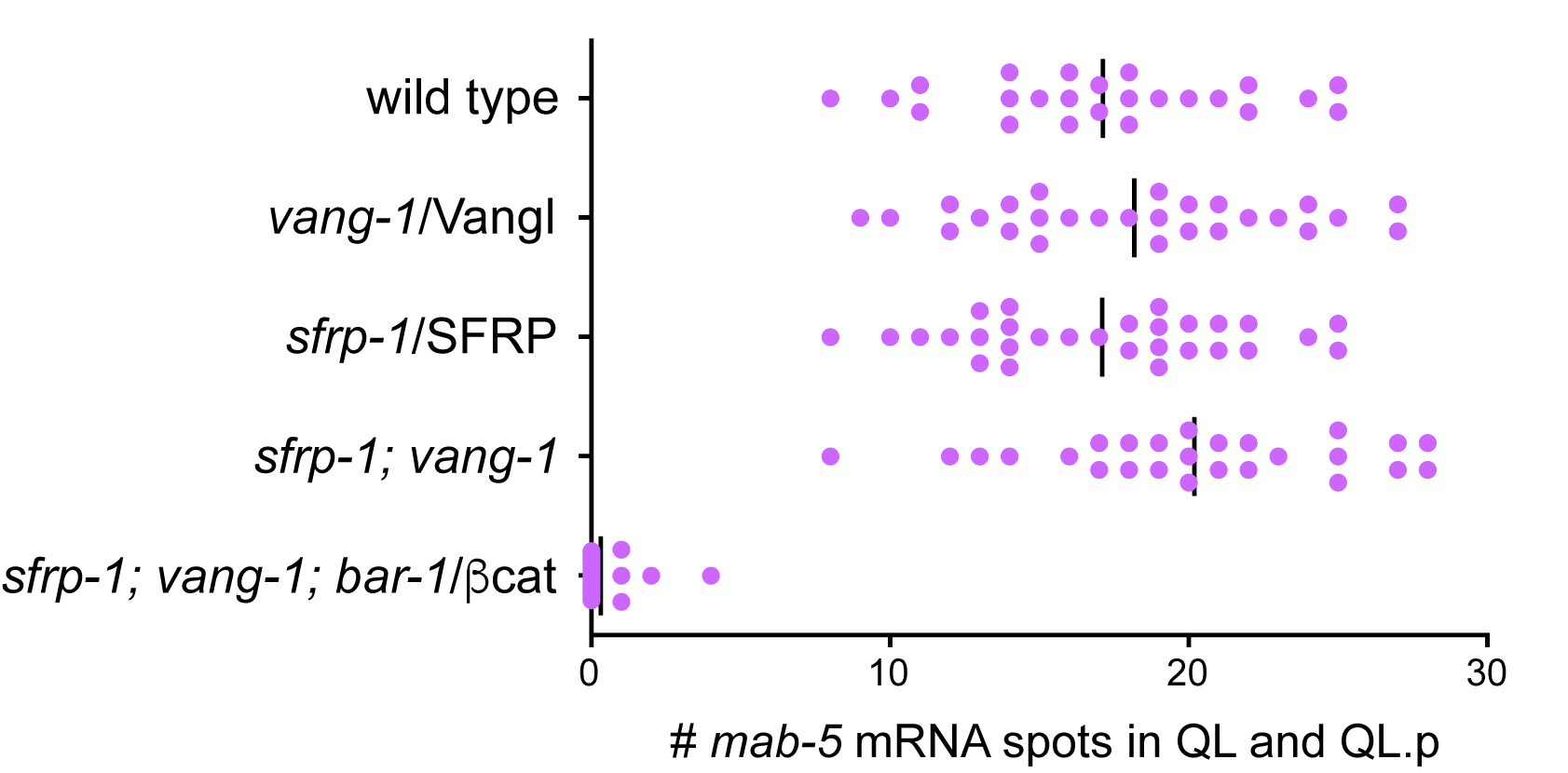

Supplement: S3 Fig — Displayed are the mab-5 mRNA counts in QL (after completion of initial migration) and QL.p. (TIF) [file pgen.1007840.s003.tif]

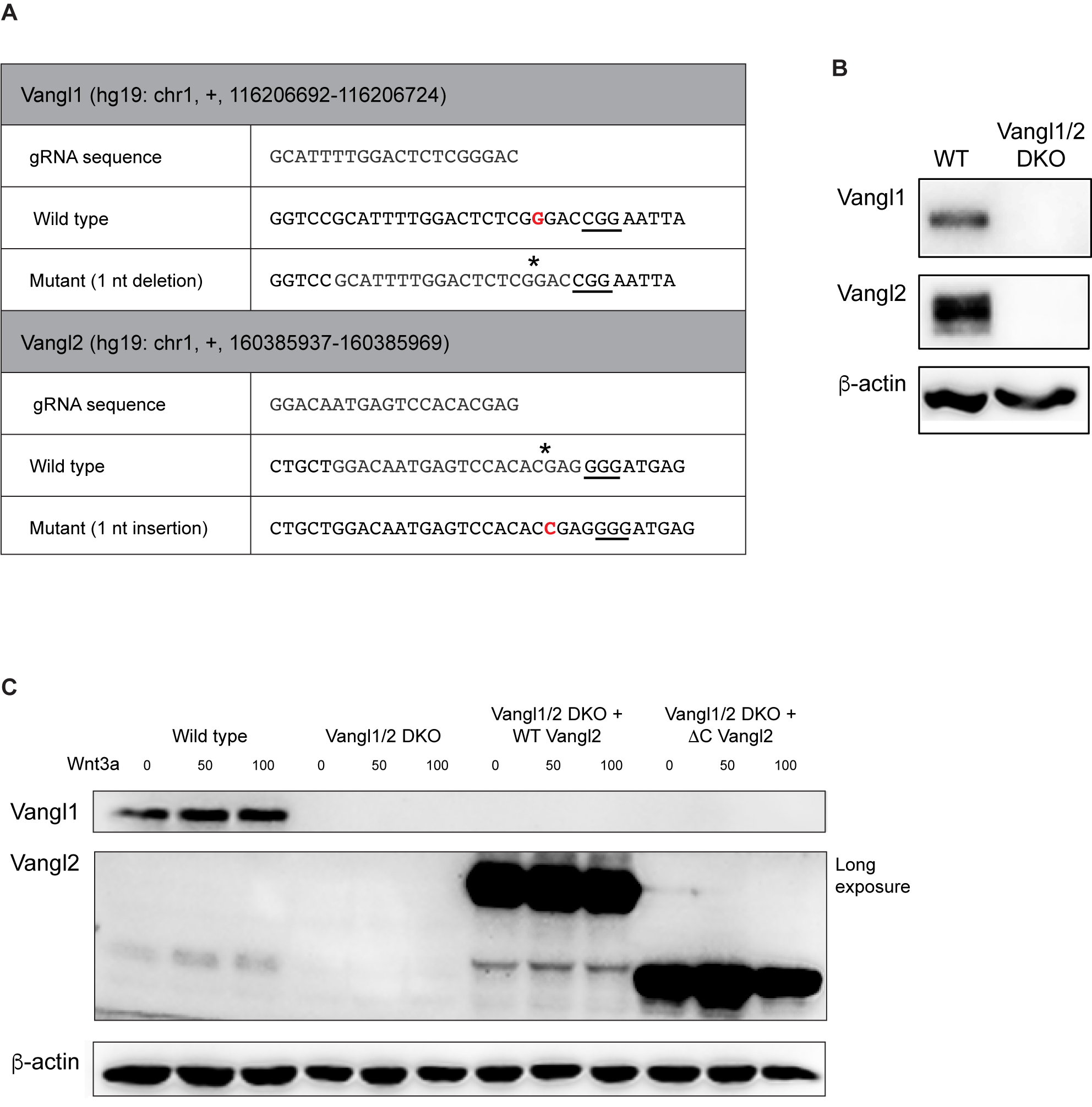

Supplement: S4 Fig — (A) Sequences of the guide RNAs and the mutations in the double mutant (DKO) cell line. (B) Western blot analysis of Vangl1 and Vangl2 expression in wild type and Vangl1/2 DKO cells. (C) Western blot analysis of Vangl1 and Vangl2 expression in control, Vangl1/2 DKO and DKO cells expressing wild type or C-terminally truncated Vangl2 (fused to EGFP). A longer exposure of Vangl2 expression is shown. (TIF) [file pgen.1007840.s004.tif]

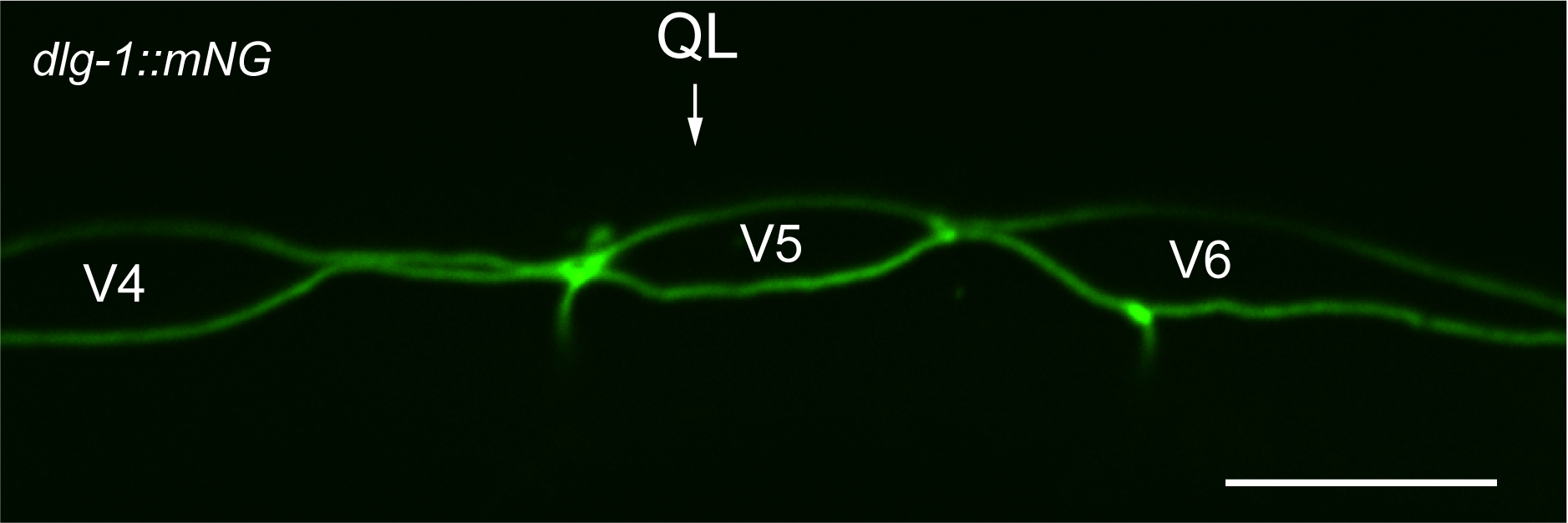

Supplement: S5 Fig — DLG-1 is expressed in the seam (V) cells, but no expression is visible in the QL neuroblast (position marked by arrow). Scale bar = 10 μm. (TIF) [file pgen.1007840.s005.tif]
